# Quantitative comparison of different iron forms in the temporal cortex of Alzheimer patients and control subjects

**DOI:** 10.1038/s41598-018-25021-7

**Published:** 2018-05-02

**Authors:** Marjolein Bulk, Louise van der Weerd, Wico Breimer, Nikita Lebedev, Andrew Webb, Jelle J. Goeman, Roberta J. Ward, Martina Huber, Tjerk H. Oosterkamp, Lucia Bossoni

**Affiliations:** 1 Department of Radiology, Leiden University Medical Center, Albinusdreef 2, 2333 ZA Leiden, The Netherlands; 2 Department of Human Genetics, Leiden University Medical Center, Einthovenweg 20, 2333 ZC Leiden, The Netherlands; 3 Percuros B. V, Leiden, The Netherlands; 4 Huygens-Kamerlingh Onnes Laboratory, Leiden University, PO Box 9504, 2300 RA Leiden, The Netherlands; 5 Department of Statistics, Leiden University Medical Center, Einthovenweg 20, 2333 ZC Leiden, The Netherlands; 6 Imperial College London, Hammersmith Hospital Campus, Du Cane Rd., London, W12 0NN England

## Abstract

We present a quantitative study of different molecular iron forms found in the temporal cortex of Alzheimer (AD) patients. Applying the methodology we developed in our previous work, we quantify the concentrations of non-heme Fe(III) by Electron Paramagnetic Resonance (EPR), magnetite/maghemite and ferrihydrite by SQUID magnetometry, together with the MRI transverse relaxation rate $({{\rm{R}}}_{2}^{\ast })$, to obtain a systematic view of molecular iron in the temporal cortex. Significantly higher values of ${{\rm{R}}}_{2}^{\ast }$, a larger concentration of ferrihydrite, and a larger magnetic moment of magnetite/maghemite particles are found in the brain of AD patients. Moreover, we found correlations between the concentration of the iron detected by EPR, the concentration of the ferrihydrite mineral and the average iron loading of ferritin. We discuss these findings in the framework of iron dis-homeostasis, which has been proposed to occur in the brain of AD patients.

## Introduction

Iron is an essential component of many enzymes and proteins that participate in a variety of biological functions in the brain, such as neurotransmitter synthesis and myelination of neurons^1^. Increases in the iron content of specific brain regions were identified in many neurodegenerative diseases, e.g. Alzheimer’s Disease (AD), Parkinson’s Disease, and Friederich Ataxia^2–6^. In AD, which is clinically characterised by progressive dementia, increased iron content in the temporal, parietal and frontal lobes has been reported^7,8^. In the presence of the pathological hallmarks of AD, iron is accumulated within and around the amyloid-beta plaques (A*β*) and neurofibrillary tangles^3,9^, mostly as ferrihydrite inside ferritin, hemosiderin, and magnetite.

The co-localization of iron with A*β* has been proposed to constitute a major source of toxicity. Indeed, *in vitro*, A*β* has been shown to convert ferric iron (Fe(III)) to ferrous (Fe(II)) iron^10,11^, which can act as a catalyst for the Fenton reaction to generate toxic free radicals, which in turn result in oxidative stress.

Mineralized forms of iron, other than ferrihydrite, are found inside^12,13^ and outside ferritin^14,15^. For example, it has been shown that magnetite and wüstite are both present in the AD brain^15^. This is of importance as such species possess Fe(II), which is associated with toxicity. Although these nanoparticles may be present in low concentrations^16^, if they are larger than 40–50 nm in diameter, they carry a permanent magnetic moment at room temperature^14,15,17^, which can significantly affect the proton Nuclear Magnetic Resonance Imaging (MRI) signal, thus allowing *indirect* imaging of iron^18–20^.

The magnetic moment of iron/iron nanoparticles dephases the proton spins of close-by diffusing water molecules, leading to the shortening of the endogenous spin-spin relaxation times (T_2_ and ${{\rm{T}}}_{2}^{\ast }$)^18,21–23^. Therefore, local iron accumulation is observed as hypointense areas on T_2_ and ${{\rm{T}}}_{2}^{\ast }$-weighted multi-echo MRI sequences. *In vivo* and post-mortem MRI have shown that in AD, cortical iron can be visualized as bands or foci of signal loss^24,25^. In addition, elevated tissue ferritin levels are often associated with low values of ${T}_{2}^{\ast }$ in AD patients^26,27^. Susceptibility-weighted imaging (SWI)^18^ and quantitative susceptibility mapping (QSM)^28,29^ are methods based on the correspondence between measured phase and local magnetic field, and were employed to assess brain iron^30,31^. However, the results from SWI analyses depend on the particular orientation, geometry and position of the dipolar field source, and both SWI and QSM do not differentiate between molecular forms of iron. Moreover, although a positive linear correlation between MRI relaxation times and tissue iron has been shown^27,30,32–35^, most of the MRI studies refer to “iron” in very general terms, while only a few studies have investigated correlations between MRI relaxation times and specific molecular iron forms, mostly as ferritin-stored iron^26,27,36^. Non-heme iron types are diverse and contribute differently to the MRI relaxation times, via their different magnetic moments^37^. Indeed, there are many other potential confounding factors in using susceptibility for absolute iron quantification, for example, a lower cell water content, abrupt susceptibility changes^18^, changes in myelination^36,38^ and co-localization with A*β*^39^.

In order to provide a systematic and quantitative view of different molecular/mineral iron forms in brain tissue, we recently developed a combination of methods^40^, by which we can quantify: (i) non-heme rhombic Fe(III); (ii) ferrihydrite (Fe_2_O_3_ ⋅ 0.5H_2_O) and (iii) magnetite/maghemite (Fe_3_O_4_/*γ*−Fe_2_O_3_). In contrast to previous studies, in which only one iron-oxide species was quantified, here we present a broad overview of iron forms obtained from a brain region where elevated iron concentration is found in AD. Furthermore, in this present work, we show how to derive the magnetic moment of magnetite and the iron loading of ferritin from magnetometry data. We apply the methodology described earlier^40^ and we complement our set of techniques with MRI, which we performed on the same tissue sample used for the other experimental techniques.

In the present study, we have focussed on the middle temporal gyrus, since the temporal lobe is the most affected in terms of AD pathological hallmarks (A*β* and *τ-*tangles), and is the earliest cortical region to develop AD pathology. Additionally, there is evidence that both AD pathological hallmarks correlate with the amount of iron and myelin accumulation in the cortex^41^.

Finally, we applied our methodology to a substantially large number of samples (22 AD patients and 14 controls) and we found significant differences in the iron forms between AD and controls. Furthermore, we show correlations, which were previously undetected, among different iron forms.

## Materials and Methods

All measurements and raw data analysis were carried out blind to diagnosis.

### MRI experiments

Formalin-fixed tissue samples of the temporal middle gyrus were obtained from the Netherlands Brain Bank (NBB). Diagnosis of AD was confirmed by neuropathological examination in agreement with the guidelines of the ethics committee of the LUMC. Patient anonymity was strictly maintained. All tissue samples were handled in a coded fashion, according to Dutch national ethical guidelines (Code for Proper Secondary Use of Human Tissue, Dutch Federation of Medical Scientific Societies).

Before the Nuclear Magnetic Resonance Imaging (MRI) experiment, the tissue was washed in phosphate buffered saline (PBS) for 24 h, to remove residual formalin and partially restore the physiological proton relaxation times to those before fixation. Finally, the tissue was inserted into a plastic tube, immersed in a proton-free solution (fomblin), and kept in place by gauze.

MRI scans were performed on a 7T horizontal bore Bruker MRI system equipped with a 23 mm receiver coil and Paravision 5.1 imaging software (Bruker Biospin, Ettlingen, Germany).

**Figure 1:**
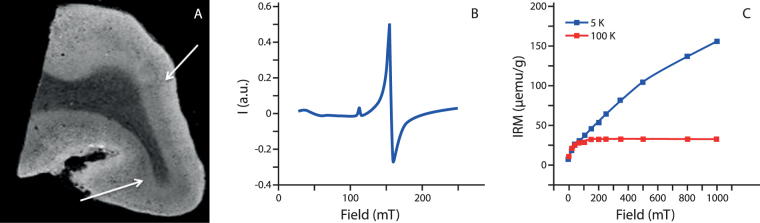
Example of raw data obtained from this study. Panel A shows the MRI scan (third echo of the Multi-Gradient-Echo scan). Some dark bands are observable in the grey matter, and indicated by white arrows. Panel B shows the smoothed EPR spectrum obtained from the same subject. Panel C illustrates the IRM data measured with the SQUID magnetometer at 100 K (red curve), showing the presence of magnetite/maghemite, and 5 K (blue curve), attributed to the presence of ferritin.

High-spatial-resolution MRI scans were obtained with a three-dimensional ${T}_{2}^{\ast }$-weighted Multi-Gradient-Echo sequence. Repetition time TR = 75 ms, echo spacing TE = 12.5, 23.3, 33.9 and 44.6 ms, 100 *μ*m isotropic resolution, flip angle = 15 degrees, 10 averages, 140 × 240 pixels. The complete imaging protocol had a total duration of 3.5 hours. The ${R}_{2}^{\ast }$ rate was obtained after fitting the signal decay with the following expression:

1 $$y=A{e}^{-TE\cdot {R}_{2}^{\ast }}$$

where *A* is a prefactor (See Supplementary Information for details on the quality of the fit). By inspecting the MRI scans of the samples from AD patients, a diffuse hypointense band, as described previously^19,25^, was observed in the grey matter (see Fig. 1A). The region of interest (ROI) selection was guided by the MRI scan: the ROI included all cortical layers in an area where the diffuse hypointense band was observed. In the absence of the diffuse hypointense band, an ROI was selected including all cortical layers. The median ${{\rm{R}}}_{2}^{\ast }$ of this ROI was calculated for each patient sample. The median was chosen instead of the mean since it was more stable with respect to the ROI selection. Analysis of the MRI data was performed with Matlab 2016.

### EPR experiments

A section of approximately 10–20 mg was dissected from the brain tissue with a ceramic scalpel, and prepared for the Electron Paramagnetic Resonance (EPR) study. Material from the same anatomical region was taken from the control subjects, for comparison. The tissue section was chosen to colocalize with the selected ROI on the MRI scan. EPR was performed with a 9 GHz spectrometer, at 12 K, according to the method presented earlier^40^. The continuous wave (cw) EPR measurements were performed using an ELEXSYS E680 spectrometer (Bruker, Rheinstetten, Germany) equipped with a rectangular cavity. The microwave frequency was 9.4859 GHz, modulation frequency 100 kHz, power attenuation 20 dB, receiver gain 60 dB and modulation amplitude 6 G_pp_. The accumulation time was 20 min. per spectrum. The concentration of Fe(III) (Fig. 1B) was determined for each patient by taking the second integral of the simulated signal, and by comparing it to the second integral of the reference sample (Fe-EDTA) of known concentration. All raw data with simulations are shown in the Supplementary Information. Data fitting to the spin Hamiltonian was performed with the EasySpin toolbox^42^. The spin Hamiltonian used for the fitting was typical for Fe(III), in the high-spin state:

2 $$H=g{\mu }_{B}({\bf{B}}\cdot {\bf{S}})+D({S}_{z}^{2}-\,\frac{S(S+1)}{3})+E({S}_{x}^{2}\,-\,{S}_{y}^{2})$$

where g is the Landé factor, *μ*_*B*_ the Bohr magneton, **B** is the applied field and **S** the spin operator. The two final terms represent the zero-field splitting, where *D* is the axial splitting, and *E* the rhombic splitting. The EPR spectra were fitted with the following parameters: S = 5/2, D = 20.96 GHz, E/D = 0.3324, g = [1.83, 1.998, 2.0151], gstrain = [0.574, 0.129, 0.0197]. More details on the spin Hamiltonian can be found elsewhere^40^.

### SQUID experiments

Subsequent to the EPR experiments, an additional tissue sample containing only/mostly grey matter adjacent to the tissue block used for MRI, was resected. This tissue sample was freeze-dried, pelleted, and studied by a Superconducting Quantum Interference Device (SQUID) magnetometer (MPMS). Isothermal Residual Magnetization (IRM) curves were measured at 100 K and 5 K^40^. We note that SQUID magnetometry is very sensitive for measuring magnetic impurities diluted in a non-magnetic host. However, one needs to make sure that the sample holder does not contain any fast-saturating component and that the field is zero and stable on the time-scale of the measurement. In this study, a Reciprocating Sample Option (RSO) probe, with sensitivity of 1 × 10^−8^ emu at low field (i.e. 0–2.5 T) was used. Despite the high sensitivity of the RSO, a few samples showed low signal-to-noise ratio (SNR) at 100 K. Those data were discarded from the analysis: we chose to set to zero the concentration of magnetite/maghemite of those samples whose 100 K-signal was close to the detection limit of the instrument, and the magnetic moment of these samples was discarded in the statistical analysis (see Results section). However, as shown in the Supplementary Information, many samples had a measurable signal and an SNR larger than 4. On the other hand, the 5 K data showed a signal that was much larger than the sensitivity of the instrument, for all samples.

The measured magnetic moment was divided by the dry mass of the sample, and the obtained IRM curve was fitted to the Langevin equation:

3 $$IRM\,[x]={M}_{s}\,f\,[\coth \,(x)-1/x]+B$$

where $x=\frac{{\mu }_{p}H}{{k}_{B}T}$, *H* is the magnetic field, *μ*_*p*_ is the average magnetic moment of the particles, *k*_*B*_ is the Boltzmann constant, *T* is the temperature, *M*_*s*_ is the saturation magnetization, *f* is the mass fraction of magnetic particles and *B* is a constant which takes into account residual fields. Both the 100 K and the 5 K data were fitted to the same Langevin equation (see Supplementary Information). IRM measured at 100 K was used to obtain the concentration of magnetite/maghemite nanoparticles and their magnetic moment, while the non-saturating data at 5 K (Fig. 1C) were used to derive the concentration of ferrihydrite^37,43,44^. In the fit, the temperature and the saturation magnetization were fixed to the experimental condition and to the literature data, respectively. For magnetite (maghemite), different values of saturation magnetization are reported, ranging from 98 (76) emu/g for bulk material^45^, to 72 emu/g for particles of size 30–40 nm^46^, to lower values for 5 nm particles^17^. Magnetite/maghemite particles with a minimum particle-size of 25–35 nm have their magnetic moment blocked at approximately 100 K^40^, therefore we used *M*_*s*_ = 84 emu/g, in the above equation as a reference value. As far as ferrihydrite is concerned, a saturation magnetization of *M*_*s*_ = 0.62 emu/g is typically accepted^43,47^.

The fraction *f* of magnetic material, the magnetic moment *μ*_*p*_, and the background constant *B* were obtained from the fit of each patient’s curve (see Supplementary Information). For ferrihydrite, the magnetic moment is related to the number of iron atoms stored within the core of ferritin, also known as the loading factor (LF). According to Néel’s theory of superparamagnetism, the relation between the two is given by^48^:

4 $${\mu }_{p}=5.92\,{\mu }_{B}\,L{F}^{\alpha }$$

where *α* = 0.5−0.6, and 5.92 *μ*_*B*_ corresponds to the ionic magnetic moment of Fe(III). Dividing LF by the maximum number of iron ions that ferritin can accommodate, i.e. 4500, we obtained the ferritin loading ratio (FLR), here expressed as a percentage, for clarity. Similarly, the magnetic moment of magnetite/maghemite is proportional to the volume of the particles. We will return to this concept in the following paragraphs.

### Statistical analysis

Statistical analysis was performed using R-Studio (R version 3.3.2). The packages used were ggplot2^49^, ggally^50^, stats^51^, outliers^52^, corrplot^53^ and ppcor^54^. All data sets were tested for normality with the methods of visual inspection, q-q plot and normal Shapiro test. Outlier detection was done by visual inspection, q-q plot and Chi-square test. Data points were excluded only when the visual inspection and the formal tests agreed with each other. If required, normality was restored upon applying a log_10_-transformation: Fe(III) concentrations have been log_10_-transformed. Fe_3_O_4_/*γ*−Fe_2_O_3_ (magnetite/maghemite) data have been transformed with the following: *log*_10_(*x* + *C*), where *x* is the particle concentration, and *C* = 100. Hypothesis testing (t-test) was performed on the transformed data. Differences in mean were considered significant when the p-value was smaller than 0.05. The variance of the distribution was also tested using the Brown-Forsythe Levene-type test, based on the absolute deviations from the median.

Spearman’s correlation coefficients for all pairs of iron forms were calculated on non-transformed data. A formal comparison between the inter-group correlations coefficients was performed by Fisher-transforming the correlations coefficients (*ρ*) and then calculating their studentized difference as a basis for a two-tailed z-test. In more detail, we transformed *ρ* into z-scores, per group (*i*)^55^:

5 $$z{r}_{i}=1/2\,\mathrm{ln}\,((1+{\rho }_{i})/(1-{\rho }_{i})).$$

Afterward, the difference in z-scores was calculated, with the following expression:

6 $${z}_{diff}=\frac{z{r}_{2}-z{r}_{1}}{\sqrt{1/({N}_{1}-3)+1/({N}_{2}-3)}},$$

where *N*_*i*_ is the number of individuals used for the calculation, and the index *i* = 1, 2 refers to the group (AD or control). This difference was used as a basis for a two-tailed z-test.

For those pairs of variables showing a significant correlation, a partial correlation test was carried out to exclude spurious correlations. In this last test, AD and control groups were pooled to increase the statistical power, in the case that the correlation comparison test was not significant.

### Sample preparation

All details of sample preparation and handling are described in our previous work^40^.

In this study, formalin-fixed material has been used. The effect of formalin fixation on the chemical state of iron is the subject of debate within the literature, with very different conclusions reached by different research groups^56^. Although frozen brain material is probably the best starting material when it comes to the quantification of metals, working with frozen brain material is not a feasible option when a comparison among different techniques performed on the same piece of tissue is carried out. Most importantly, MRI cannot be easily done on frozen tissue, due to the very short T_2_ values of frozen tissue as well as the long duration of high-resolution scans (typically of a few hours), which may affect the integrity of the tissue.

Finally, from a methodological point-of-view, it is worth noting that our results have been obtained without the need of chemical purification of the different iron forms, nor by performing any biochemical assay test, being based purely on the magnetic properties of the tissue.

## Results

Table 1 reports the characteristics of the individuals and the concentration of the measured iron forms, as means and standard deviations. P-values obtained from the t-test (mean comparison) are also reported. No significant differences were found in age or gender between the AD and control group.

**Table 1:** Summary of sample characteristics and experimental results.

	AD (n = 22)	Control (n = 14)	p-value mean test
*Characteristics*			
Male	8	4	0.248
Female	14	10	0.414
Mean age (range, yrs)	76.7 (43–96)	79.8 (64–91)	0.673
*Experimental Results*			
${{\rm{R}}}_{2}^{\ast }$ (mean ± sd, ms^−1^)	0.033 ± 0.005	0.028 ± 0.004	0.008
Fe(III) (mean ± sd, *μ*g/g_*ww*_)	6.74 ± 3.48	5.29 ± 3.66	0.125
Fe_3_O_4_/*γ*−Fe_2_O_3_ (mean ± sd, ng/g_*dw*_)	118.83 ± 125.87	121.03 ± 123.11	0.878
Fe_2_O_3_ • 0.5H_2_O (mean ± sd, *μ*g/g_*dw*_)	381.12 ± 178.97	240.84 ± 98.02	0.007

A chi-square test was used to assess differences in gender between AD and control group. A Mann-Whitney U test was used to assess differences in age between AD and control group. Student’s t-test was performed to test differences in the means of the measured iron concentrations. Statistical tests were repeated with and without the presence of the outliers, and the same conclusion about the significance of the test was reached. The index *dw* refers to dry weight, and *ww* refers to wet weight.

The value of ${R}_{2}^{\ast }$ was significantly higher in the AD group vs. the control group (p = 0.008). Ferrihydrite concentration was significantly higher in the AD group (p = 0.007), while the ferritin loading ratio (FLR) was not significantly different between groups (p = 0.362). The concentration of magnetite/maghemite (note that the magnetite/maghemite will be named “magnetite” in the figures, for simplicity) nanoparticles was not different between the two groups (p = 0.878), while the average magnetic moment of the particles was larger in the AD group (p = 0.002). Finally, the Fe(III) concentration was not significantly different between groups (p = 0.125).

The extent to which the variance between the two groups is identical was also independently tested. We found that only the distribution of ferrihydrite concentration showed a significantly different variance between AD and control group (p = 0.026). The results of this test are reported in the Supplementary Information.

**Figure 2:**
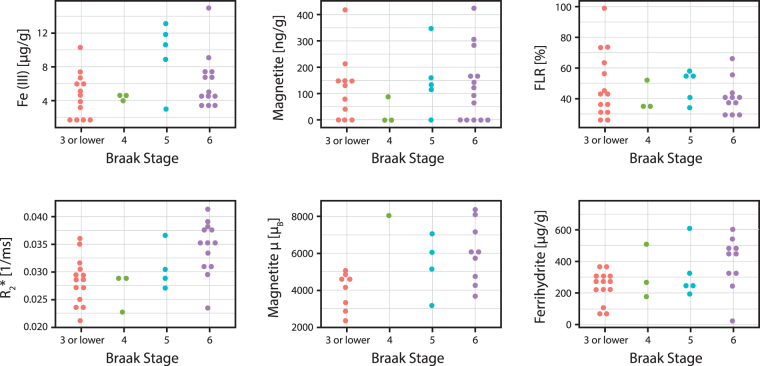
Visual representation of the iron forms grouped by Braak Stage. Each plot represents the concentration/magnetic property of the different iron forms grouped by the Braak Stage-number (outliers have been removed). All controls with Braak Stage equal to 3 or lower have been grouped in the variable “Braak Stage 3”. The AD patients have Braak Stage varying from 4 to 6. The *μ* symbol refers to the magnetic moment of the magnetite particle. For missing data in the ‘Magnetite *μ*′ variable, please see Materials and Methods.

Subsequently, the individuals were stratified by their Braak Stage: control individuals with Braak Stage equal to or lower than 3 were grouped together. We found that those iron forms showing a significant inter-group difference in the mean, i.e. ${R}_{2}^{\ast }$ values, ferrihydrite concentration and magnetite/maghemite magnetic moment, also showed a positive trend with increasing Braak Stage (Fig. 2), although no formal test was done due to the limited number of samples in each group.

**Figure 3:**
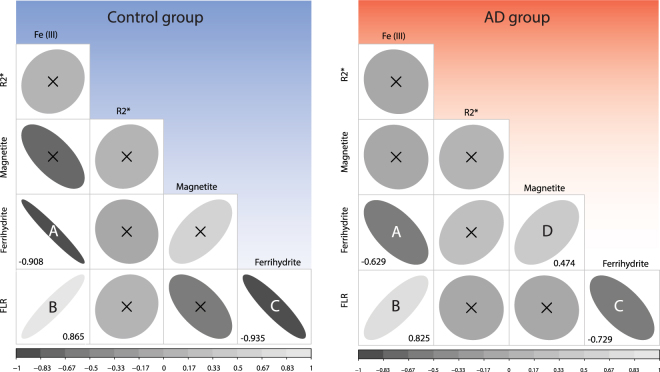
Correlogram of the iron forms measured in this study and grouped by diagnosis. The ellipse eccentricity and color intensity are proportional to the Spearman’s correlation coefficient for a specific pair of iron forms (see also scale bar at the bottom). Left panel refers to the control group (N = 11, after outliers were removed as explained in the Materials and Methods section) while the right panel refers to the patients group (N = 18, after outlier removal). Correlograms which are not statistically significant, i.e. p-value > 0.05, are crossed. For those correlograms with p-value < 0.05, the correlation coefficient is reported in the respective squares. Letters are used to facilitate the comparison between pairs of variables.

We further carried out a correlation study to investigate associations among the measured iron forms, and between iron and ${R}_{2}^{\ast }$ values. Figure 3 represents the correlation matrices (named correlogram hereafter) for all measured iron forms, in the form of ellipses, the eccentricity and intensity of which is equal to the Spearman’s correlation coefficient between the variables reported on the axes.

Our initial findings indicate that some iron forms strongly correlate with each other. In particular, Fe(III) concentration positively associates with the FLR (Spearman’s *ρ* = 0.825 for the AD patients, and 0.865 for the control subjects) and negatively with ferrihydrite concentration (*ρ* = −0.629 for the AD, and −0.908 for the control group). Additionally, ferrihydrite concentration correlates negatively with the FLR (*ρ* = −0.729 for the AD, and −0.935 for the control group), and the magnetite concentration associates positively with ferrihydrite concentration (*ρ* = 0.474) only for the AD group. The rest of the correlations were not significant.

When we compared the correlation coefficients between the two groups (AD vs. controls), we found that they were not significantly different, although the ferrihydrite-Fe(III) and the FLR-ferrihydrite pairs were associated with a rather small p-value (0.076 and 0.079, respectively) (see Supplementary Information, Table SII).

Since three variables were mutually correlated, we performed a partial correlation test to assess the correlation between two variables, while controlling for the effect of the third one. The results of the partial correlation are reported in Table 2. Note that during this test, the AD and control data were pooled, in the light of the results of the correlation comparison test.

**Table 2:** Result of the partial correlation test.

	Fe(III)	Ferrihydrite	FLR
Fe(III)	1	−0.033	0.700
p-value	0	0.852	3.453 • 10^−5^ (***)
Ferrihydrite		1	−0.464
p-value		0	1.279 • 10^−2^ (*)
FLR			1
p-value			0

The partial correlation coefficient is reported together with the p-value of the test. AD and control groups have been pooled. The asterisk (*) indicates that the p-value is less than 0.05 while the triple asterisk (***) indicates that p < 0.001.

The partial correlation test shows that the negative correlation found between Fe(III) and ferrihydrite may be spurious, and can be explained by the effect of the FLR variable.

## Discussion

In this study, we seek to identify the differences in iron forms in the temporal cortex of Alzheimer patients and age- and gender-matched controls. A unique combination of techniques^40^ enables us to differentiate these iron forms to a much larger degree than previously possible. One brain region affected by Alzheimer’s disease, the temporal cortex, was selected from different AD patients and control subjects, and an extensive statistical analysis was employed. The temporal lobe was chosen since it is the most affected region in terms of AD pathological hallmarks (A*β* and *τ*-tangles), as mentioned earlier. In order to investigate different iron forms on the same sample, experiments were performed by different techniques, which required the use of formalin-fixed tissue (see Materials and Methods).

In the following discussion, we first analyse each iron form separately, and then summarize the statistically relevant conclusions which we divide into correlations common to both groups, and differences between groups. Additionally, we compare these findings with MRI ${{\rm{R}}}_{2}^{\ast }$ values obtained from the very same anatomical region.

The g′ = 4.3 EPR band was found in every measured sample. This signal has been often ascribed in literature to non-heme high-spin Fe(III) in a system of rhombic symmetry^57,58^. We exclude the possibility that this signal is due to sample environment contamination since it was absent when either the empty cavity or a test tube containing the fixating solution were studied. The signal can be ascribed to the m_*s*_ = ±3/2 Kramers doublet of rhombic S = 5/2 systems and it is not fully understood^59^. This iron form has taken different names in the literature, including mono-nuclear “spurious” iron, labile iron, iron bound to complexes of low molecular weight, and non-transferrin bound iron (NTBI)^1,60^. It is beyond the scope of this work to identify the specific ligand environment responsible for the observed signal. However, in our previous work, we already discussed why this signal is not ascribable to transferrin^40^, the main iron transporter protein^61^.

The concentration of Fe(III) in the temporal cortex found in this study ranged between 1.58 and 14.95 *μ*g/g (wet weight), equivalent to 28.3 *μM* and 267.6 *μM* respectively, while the concentration of intracellular labile iron pool should fall in the 0.2–10 *μ*M range, as assessed by several studies, which used EPR or fluorescent probes to quantify labile iron in homogenized tissue, cells, and ferritin solution^62–66^. A quantitative comparison between the iron concentration obtained in our study versus the commonly accepted values suggests that our g′ = 4.3 band cannot *exclusively* be attributed to the labile iron pool. On the other hand, here we speculate that the signal can originate from a site that is part of the ferritin iron core, but does not show superparamagnetic properties. Indeed, this signal is often found in studies of the incorporation of iron into mammalian and bacterial ferritins^67,68^. Additionally, from a magnetic viewpoint, this iron could be present on the outer surface of the core, thus experiencing a smaller Weiss molecular field than those atoms strongly bound to the core. This may result in a lower Néel temperature, as already proposed^69^, and the effective paramagnetic behaviour demonstrable by the g′ = 4.3 signal^40^.

The ferrihydrite concentrations assayed in the present study (range: 25.8–730.7 *μ*g/g) are in agreement with those reported earlier for ferritin^18^. Ferrihydrite concentration provides a measure of the ferritin-bound iron in the tissue, while its magnetic moment is related to the iron loading of the protein. Typically, 4500 iron atoms per protein are considered to be the maximum which the core can store *in vitro*. However, *in vivo* loading may be lower: some studies reported a loading of 1500 atoms *in vivo*^70,71^, corresponding to roughly 33% of the protein capacity. Other findings show that, when ferritin is fractionated by density gradient centrifugation, the full range of ferritins, from native apoferritin to full ferritin, is found^72,73^. Our control subjects display an FLR ≤50% (the percentage refers to a maximum loading of 4500 atoms), while the AD patients display larger ferritin filling ratios, although this difference is not statistically significant.

Another mineralized iron form we were able to detect is magnetite/maghemite. Our IRM measurements are not able to disentangle magnetite and its oxidation product, maghemite. The concentration of magnetite/maghemite assayed (range: 0–418.4 ng/g) and the particles’ size that we previously estimated^40^ are within the range reported by others for magnetite in the frontal and temporal lobe^15,16^, although here we are sensitive to larger particles than in previous studies (see Materials and Methods). The magnetic moment (*μ*) of these particles is a measure of their volume. We point out that, given the large magnetic moment associated with these particles, i.e. larger than 100 *μ*_*B*_ for ferrihydrite and 1000 *μ*_*B*_ for magnetite/maghemite, this study suggests that MRI could be employed to selectively identify them in the brain of patients with AD.

**Figure 4:**
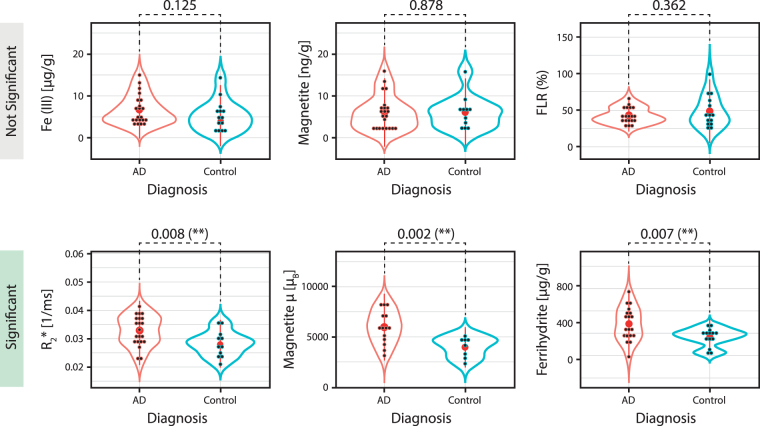
Violin plots of the measured iron forms and their magnetic properties. Experimental data, i.e. iron form concentrations and magnetic moment, grouped by diagnosis (AD and control). Each plot shows individual data (black dots), the mean (red dot) and the histogram of the data (curve line). The number on top of the data is the p-value obtained from the Student’s t-test performed on the two groups. “Significant” indicates a p-value less than 0.05. The top row represents the iron forms and property showing a not significant result, while the bottom row represents the significantly different iron forms. The double asterisk (**) signifies that the p-value is less than 0.01.

In the following, we discuss the differences in the above-described iron forms between AD and control subjects. From Fig. 4, we observe that ferrihydrite levels, magnetite/maghemite magnetic moment and ${{\rm{R}}}_{2}^{\ast }$ values are elevated in the temporal cortex of AD patients. The same iron forms show a positive trend with the Braak Stage (Fig. 2), which describes the distribution pattern of neurofibrillary changes^74^ and reflects the clinical progression of AD. This is in agreement with the work of Van Duijn *et al*., who recently showed a correlation between iron accumulation in the frontal cortex, the amount of A*β* and *τ* pathology^41^ and the Braak Stage.

The increase of ferrihydrite levels in the absence of an increase in the average FLR confirms earlier studies, suggesting a local accumulation of iron in the brain of AD patients^2^ and a higher expression of ferritin protein^71^. Given the recent observation that ferritin levels in the cerebrospinal fluid and in the plasma are associated with cognitive impairment progression in AD and with amyloid burden^75,76^, and given the results of the present study, we believe that more investigations on the role of ferritin in AD progression are needed.

We did not find higher concentrations of magnetite/maghemite in the brain region investigated by us (middle temporal lobe). However, we did observe larger magnetic moments which suggest that magnetite/maghemite particles found in the AD tissue are larger than those found in controls (see Fig. 4). To explain higher magnetite levels, it was hypothesized that biogenic magnetite could originate from malfunctioning ferritin^9,14,16^, and more recently, A*β* was shown to mediate the formation of magnetite from ferrihydrite *in vitro*^10^. However, a few recent studies show that A*β* is able to catalise magnetite formation, even in absence of ferritin, under non-physiological pH conditions^77^. Additionally, Maher *et al*.^78^ reported that some of these magnetite particles, which were found in the frontal cortex, had a characteristic spherical, rather than cubic, geometry and shared a striking resemblance to anthropogenic, combustion-derived magnetite nanoparticles. Leaving aside the origin of these anthropogenic/biogenic nanoparticles, our results suggest that the size of these magnetite particles is different in presence of AD when compared to the control case.

The elevated ${R}_{2}^{\ast }$ values in the AD subjects could be caused by an increase of cortical iron, as suggested by previous *ex vivo*^79^ and *in vivo*^80^ observations, where cortical tissue was studied with the same ${R}_{2}^{\ast }$-weighted pulse sequence. Indeed, QSM suggests that an increase in the susceptibility of the neocortex is associated with local iron accumulation^28,81,82^, possibly increasing with the disease staging. Additionally, a significant correlation was found between MRI pixel intensity, iron and myelin^25^. However in the present study, we did not observe a correlation between the ${{\rm{R}}}_{2}^{\ast }$ values and any assessed iron form. This may be due to the small dynamic range of iron concentration within the temporal cortex, which can affect the Spearman’s correlation coefficient, or to the difference in the spatial resolution of the different techniques (see Materials and Methods). An ${{\rm{R}}}_{2}^{\ast }$ increase may also originate from susceptibility changes occurring in tissue lesions, i.e. A*β* and *τ*-tangles^83^, as pointed out by the trend between ${R}_{2}^{\ast }$ and Braak Stage shown in Fig. 2, or be caused by microhemorrhages, or myelin changes^36,38^ also taking place in AD^25^.

Finally, our correlation study (see Table 2) indicates that the Fe(III)-form quantified by EPR is not ‘unspecific’, but it is most likely associated with the iron loading of ferritin. This finding suggests that the EPR-detected iron could originate from the iron pool on the outer-surface of the ferrihydrite mineral, as suggested above. Additionally, the surprising negative correlation between ferrihydrite concentration and the FLR may be understood in a scenario in which, when the cytoplasmic iron pool increases, ferritin over-expression is initiated as a protective mechanism^6^. This can start a process in which iron is rapidly taken up by newly expressed ferritins, thus resulting in a heterogeneous FLR, and in ferritins which are on average less-filled than those found under normal iron levels. Ultimately, the moderate correlation between magnetite/maghemite and ferrihydrite is intriguing and deserves future investigation.

In this work, the temporal cortex of AD patients (N = 22) and control subjects (N = 14) was investigated to study the different molecular forms and magnetic properties of iron. An indirect assessment of the iron concentration was performed by measuring the ${{\rm{R}}}_{2}^{\ast }$ in the gray matter. The same region was also investigated by EPR and SQUID magnetometry and the concentration of non-heme rhombic Fe(III), magnetite/maghemite and ferrihydrite, and their respective magnetic moments were derived. Statistical analysis revealed that ${{\rm{R}}}_{2}^{\ast }$ values, ferrihydrite levels, and magnetite/maghemite magnetic moment are elevated in the temporal cortex of AD patients. This suggests a local accumulation of iron and an interaction between magnetite and, possibly A*β in vivo*, in agreement with previous studies.

The correlation study of the measured iron forms indicates that Fe(III) strongly correlates with the amount of iron stored within ferritin, and the negative associations between ferrihydrite and the FLR could be interpreted as a result of heterogeneously-loaded ferritins, possibly due to ferritin over-expression, to cope with iron overload.

## Electronic supplementary material

Supplementary Information
