# Supplementary material for: Quantitative comparison of different iron forms in the temporal cortex of Alzheimer patients and control subjects
Source: Sci Rep. 2018 May 2;8:6898. doi: 10.1038/s41598-018-25021-7 (PMC5932027; doi:10.1038/s41598-018-25021-7)
Supplement: Supplementary file 1 — Supplementary Information [file 41598_2018_25021_MOESM1_ESM.pdf]

SUPPLEMENTARY INFORMATION

Quantitative comparison of different iron forms in the temporal cortex of Alzheimer patients and control subjects

Marjolein Bulk[1,2,3], Louise van der Weerd[1,2], Wico Breimer,[4] Nikita Lebedev,[4] Andrew Webb,[2] Jelle Goeman,[5], Roberta J. Ward,[6] Martina Huber,[4] Tjerk H. Oosterkamp,[4] and Lucia Bossoni[1,4]

[1] *Department of Radiology, Leiden University Medical Center, Albinusdreef 2, 2333 ZA Leiden, The Netherlands*

[2] *Department of Human Genetics, Leiden University Medical Center, Einthovenweg 20, 2333 ZC Leiden, The Netherlands*

[3] *Percuros B. V., Leiden, The Netherlands*

[4] *Huygens-Kamerlingh Onnes Laboratory, Leiden University, PO Box 9504, 2300 RA Leiden, The Netherlands*

[5] *Department of Statistics, Leiden University Medical Center, Einthovenweg 20, 2333 ZC Leiden, The Netherlands and*

[6] *Imperial College London, Hammersmith Hospital Campus, Du Cane Rd., London W12 0NN, England*

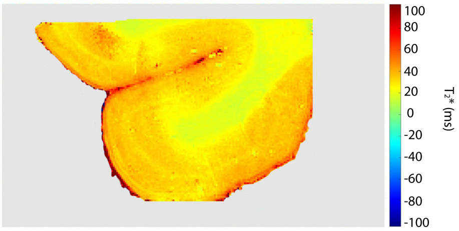

(a) MRI T_2^* map obtained from an MGE scan.

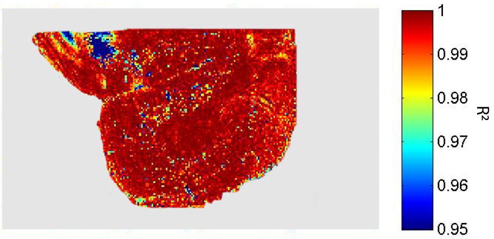

(b) Coefficient of determination (R^2) of the fit.

Figure S 1: Example of a T_2^* map obtained from one of the studied subjects, by fitting the Multi-Gradient-Echo (MGE) scan signal to Equation (1) of the main manuscript (panel **a**). The quality of the fit is expressed as the coefficient of determination (R^2, panel **b**). Notice the threshold between 0.95 and 1. This figure indicates the accuracy of the model used to obtain the fitted T_2^* values. A mask has been applied to the background for illustrative purposes.

Table S I: Summary of the Levene test for variance. A Brown-Forsythe Levene-type test based on the absolute deviations from the median was used to assess inter-group differences in variance among the iron forms and magnetic properties presented in this study. Only the ferritin concentration shows a significant difference in the variance test. A p-value <0.05 is considered significant. Fe_3O_4 refers to magnetite; $\gamma-Fe_2O_3$ refers to maghemite and $Fe_2O_3 \cdot H_2O$ refers to ferrihydrite.

**Iron form and property**	**p-value**
R_2^*	0.1769
Fe(III)	0.1902
$Fe_3O_4/\gamma-Fe_2O_3$ concentration	0.6624
$Fe_3O_4/\gamma-Fe_2O_3$ magnetic moment	0.2213
$Fe_2O_3 \cdot H_2O$ concentration	0.0261
$Fe_2O_3 \cdot H_2O$ FLR	0.0581

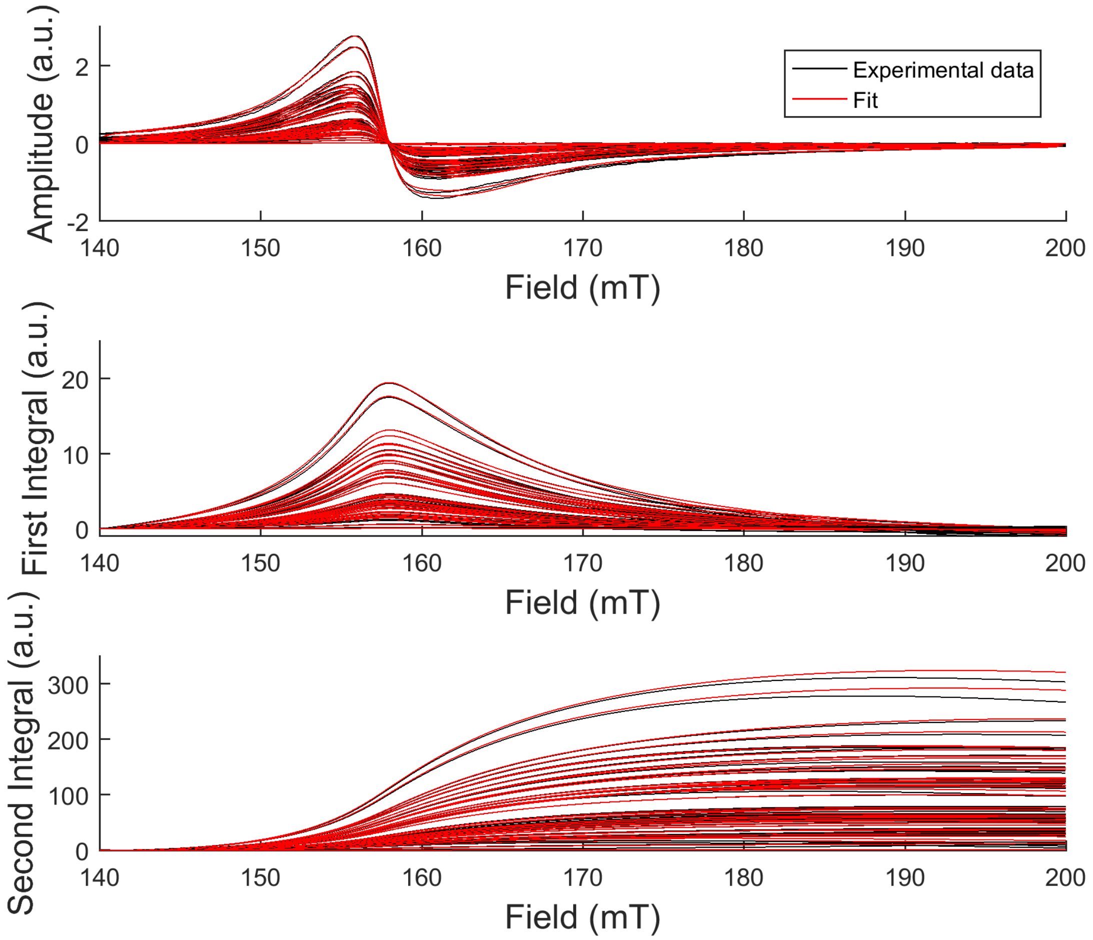

Figure S 2: Raw EPR data and fit. The raw EPR data (top panel), first integral (middle panel) and second integral (bottom panel) are shown in black. The red lines are the fits to the spin Hamiltonian previously described [1]. All 36 samples are shown.

[1] P. Kumar, M. Bulk, A. Webb, L. V. D. Weerd, H. Tjerk, M. Huber, and L. Bossoni, Scientific Reports **6**, 1 (2016).

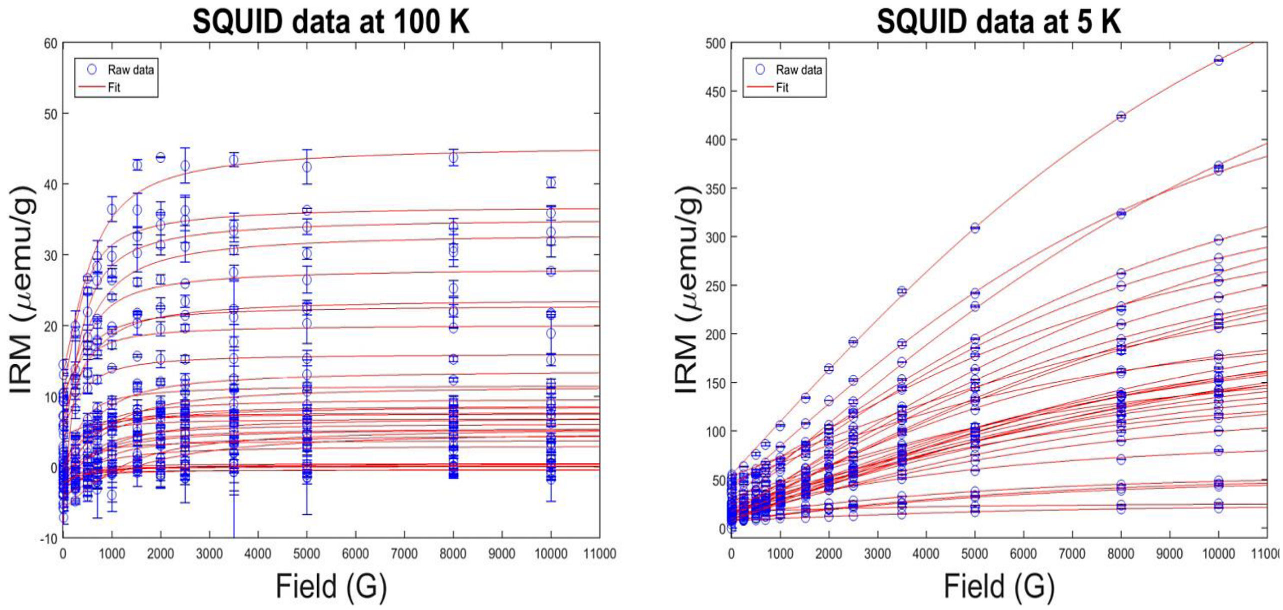

Figure S 3: Raw SQUID data and fits. SQUID data measured at 100 K (left panel) and at 5 K (right panel) are shown in blue. Error bars are also displayed. The fit to the Langevin curve is shown in red. All 36 samples are shown.

Table S II: Results of the correlation coefficient comparison. The AD samples size (N) used for this comparison was 18, while the control sample size was 11. ρ indicates the Spearman's correlation coefficient. The p-values were obtained from a two-sided test statistical distribution (See Materials and Methods on the manuscript for explanation).

**Pair of variables**	**AD**<br>ρ, N	**C**<br>ρ, N	z_{diff}	**p-value**
**Ferritin - Fe(III)**	-0.629, 18	-0.908, 11	1.77	0.076
**FLR - Fe(III)**	0.825, 18	0.865, 11	0.33	0.748
**FLR - Ferritin**	-0.729, 18	-0.935, 11	-1.759	0.079
**Magnetite - Ferritin**	0.474, 18	0.49, 11	-0.048	0.962

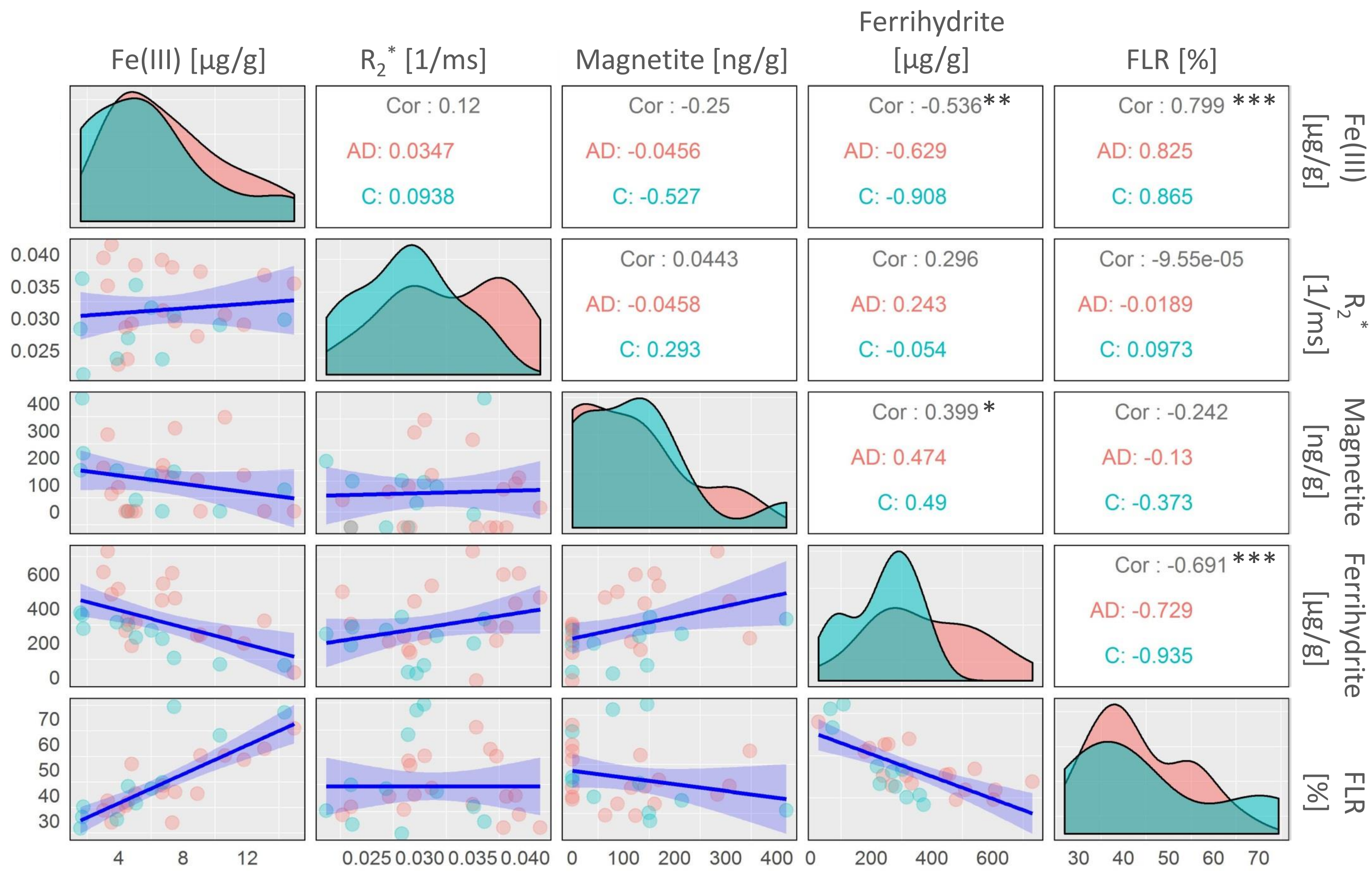

Figure S 4: Correlation plot of the forms of iron measured in this study. Each iron form is plotted against the other. The diagonal line of plots represents the histograms, the bottom-left diagonal shows a scatter plot of the raw data (outliers have been removed). Red points refer to the AD group, the blue points refer to the control group. The thick solid blue line represents linear fits to the pooled data (i.e. not stratified by diagnosis). The top-right diagonal shows the Spearman's correlation coefficient for the pooled data ("Cor"), the AD group ("AD"), and the Control group ("C"). The asterisks on top of the number indicate the p-value, according to the following convention: "*" indicates p-value < 0.05; "**" indicates p-value < 0.01 and "***" indicates p-value < 0.001. The labels on top of the plots refer to the different iron forms measured (see main manuscript). The shadow represents the 95% confidence level interval of the linear regression model. The units of measurement of the iron concentrations are indicated in square brackets.
